# The Phenotype of *Physcomitrium patens SMC6* Mutant with Interrupted Hinge Interactions

**DOI:** 10.3390/genes16091091

**Published:** 2025-09-16

**Authors:** Karel J. Angelis, Marcela Holá, Radka Vágnerová, Jitka Vaculíková, Jan J. Paleček

**Affiliations:** 1 Institute of Experimental Botany, Czech Academy of Sciences, Rozvojová 263, 165 00 Prague, Czech Republic; 2 National Center for Biomolecular Research, Faculty of Science, Masaryk University, Kamenice 5, 625 00 Brno, Czech Republic; 3 Mendel Centre for Plant Genomics and Proteomics, Central European Institute of Technology, Masaryk University, Kamenice 5, 625 00 Brno, Czech Republic

**Keywords:** *Physcomitrium patens* SMC5/6, hinge domain, protein–protein interactions, DSB repair, mutagenesis, gene targeting, rDNA stability, protonemata development

## Abstract

**Background/Objectives**: The Structural Maintenance of Chromosomes (SMC) proteins form essential heterocomplexes for the preservation of DNA structure and its functions, and hence cell viability. The SMC5/6 dimer is assembled by direct interactions of ATP heads via the kleisin NSE4 bridge and by SMC hinges. The structure might be interrupted by a single point mutation within a conserved motif of the SMC6-hinge. We describe the phenomena associated with the impairment of the SMC5/6 complex with morphology, repair of DNA double strand breaks (DSB), mutagenesis, recombination and gene targeting (GT) in the moss *Physcomitrium patens* (*P. patens*). **Methods**: Using CRISPR/Cas9-directed oligonucleotide replacement, we have introduced two close G to R point mutations in the hinge domain of SMC6 of *P. patens* and show that both mutations are not toxic and allow viability of mutant lines. **Results**: The *G514R* mutation fully prevents the interaction of SMC6 not only with SMC5, but also with NSE5 and NSE6, while the mutation at *G517R* has no effect. The *Ppsmc6_G514R* line has aberrant morphology, spontaneous and bleomycin-induced mutagenesis, and maintenance of the number of rDNA copies. The most unique feature is the interference with gene targeting (GT), which is completely abolished. In contrast, the *Ppsmc6_G517R* line is close to WT in many aspects. Surprisingly, both mutations have no direct effect on the rate of DSB repair in dividing and differentiated cells. **Conclusions:** Abolished interactions of SMC6 with SMC5 and NSE5,6 partners, which allow DSB repair, but impair other repair and recombination functions, suggests also regulatory role for SMC6.

## 1. Introduction

The SMC6 (structural maintenance of chromosome 6) protein is a component of the highly conserved SMC5/6 complex that is composed of SMC5 and SMC6 heterodimers and six non-SMC elements, NSE1-6. The core *SMC* and *NSE1-4* subunits are essential genes in most organisms studied so far [1].

SMC proteins share a common architecture, globular subdomains of a Walker ATPase, associate to form the ‘head domain’ by combining the N- and C-termini of folded SMC molecule. The two halves of the head are connected by a long anti-parallel coiled-coil ‘arm’ and capped by a ‘hinge’ domain where the coiled-coils reverse direction. Two SMC molecules then form dimers [2].

Connected SMC5 and SMC6 molecules form a stable SMC5/6 heterodimer complex through the interaction of hinge domains on one end [2] and connecting ATPase heads by the kleisin-KITE subcomplex (NSE1,3,4) [1,3,4] on the other. The association of the SMC head domains upon ATP binding and hydrolysis can dynamically regulate the opening and closing of the SMC complex ‘ring’ [5], while single-strand DNA (ssDNA) binding at the hinge [6,7] promotes conformational changes leading to DNA loading through the heads of SMC complexes [8,9].

Sergeant et al. [10] showed that *Schizosaccharomyces pombe* (*S. pombe*) *Smc5* and *Smc6* interact through their hinge domains and that a temperature-sensitive mutant of *Rad18* (*Smc6*) is mutated at the glycine residue G551R in the hinge region of the unique conserved domain PPxGPxG motif. This mutation affects SMC6 protein fold [2] and, therefore, abolishes the interactions between the hinge regions of Rad18 (Smc6) and Spr18 (Smc5) [10]. In *P. patens SMC6* locus, this residue is at position aa514. In addition to the interaction of SMC6 with the SMC5 hinge and NSE4, we have recently described an interaction with two other SMC5/6 subunits, NSE5 and NSE6 [11,12]. Both NSE5 and NSE6 subunits interact at the head-proximal arm region of SMC6 and the mid-arm region of SMC5, and NSE5 also binds the hinge-proximal arm of SMC5.

The SMC5/6 still plays enigmatic roles in the response to DNA damage and its repair. Crucial for performing its functions is the formation and maintenance of a dimer structure. The association of SMC5 and SMC6 is achieved through the interaction of hinge regions of SMC subunits on one end, and on the other, by bridging of ATPase heads by the NSE1, NSE3 and kleisin NSE4 subcomplex [3]. In addition to these canonical connections, specific NSE5 and NSE6 subunits crossbridge SMC arms [11,12]. For the dissection of kleisin bridging of SMC5-SMC6, we previously induced a mutant phenotype of the moss *P. patens* by attenuating the transcription of the *SMC6* and *NSE4* genes by targeted binding of nonfunctional Cas (dCas9) nuclease [13].

In this study, we go further, to dissect the effect of the modulation of the SMC5/6 structure by uncoupling the SMC5-SMC6 interaction at the hinge. We induced the *SMC6 G514R* (*SpG551R*) mutation, critical for hinge interaction, to dissect the impact of SMC5 and SMC6 hinge uncoupling on morphogenesis, bleomycin sensitivity, mutagenesis, genome stability, DSB repair, and GT in *P. patens*.

## 2. Materials and Methods

The methods of *P. patens* cultivation, construction and cloning of mutants, as well as an analysis of various aspects of their phenotype, were published in detail in previous papers [11,12,13,14,15].

### 2.1. Plant Material and Cultivation

The wild-type (WT) *P. patens*, accession (Hedw.) B.S.G. [16], was used as a source material for mutant lines. Moss lines were maintained either as a ‘spot inocula’ on BCD agar medium [14] enriched with 1 mM CaCl_2_ and 5 mM ammonium tartrate (BCDAT medium), or propagated as moss lawns on Petri plates with BCDAT agar overlaid with cellophane disks by a weekly subculture of homogenized tissue in growth chambers at a 18/6 h day/night and in a 22/18 °C temperature cycle [14,17]. For the observation of caulonema growth, the Petri plates with BCDAT medium supplemented with 0.5% sucrose and 1.5% agar were cultivated vertically [18].

A one-day-old (1 d) protonema culture for DNA repair experiments was prepared from a one-week-old (7 d) protonema lawn on Petri plates. Scraped tissue was suspended in BCDAT medium, sheared at 10,000 rpm for two 1 min cycles with a T25 homogenizer (IKA, Staufen, Germany), and left to recover as a liquid culture for 24 h in a cultivation chamber with gentle shaking at 100 rpm. This treatment yielded a suspension of 3–5 cell filament fragments with up to 50% of apical dividing cells, which provides a unique system for studying dividing plant tissue.

### 2.2. Treatments and Sensitivity Assay

DNA DSBs were introduced with freshly prepared solutions of bleomycin sulfate supplied as Bleomedac inj. (Medac, Hamburg, Germany). Protonemata were dispersed in liquid BCDAT medium containing bleomycin and treated for 1 h. After the treatment, recovered and washed tissue was inoculated as eight explants per quadrant in a Petri plate with drug-free BCDAT agar without cellophane overlay and left to grow. The effect of treatment was evaluated after 3 weeks by weighing explants. The fresh weight of the treated explants was normalized to the fresh weight of untreated explants of the same line and plotted as % of ‘Relative fresh weight’. Every experiment was carried out twice with three technical replicates and statistically analyzed by Student’s *t*-test.

### 2.3. Spontaneous and Induced Mutagenesis

Mutagenesis was assessed using the adenine phosphoribosyl transferase (*APT*) gene reporter system [19]. Loss of function in the *APT* gene confers resistance to a toxic adenine analog, 2-fluoro adenine (2FA). We used a 1 d untreated or bleomycin-treated protonema culture, of which 1 mL was used to determine tissue dry weight, while the remaining culture was planted onto BCDAT medium supplemented with 5 µM 2FA and overlaid with cellophane disks in Petri plates. After 3 weeks of incubation with two cellophane transfers to fresh plates, the 2FA-resistant colonies (2FA^R^) were counted. The frequency of mutagenesis is given as the number of 2FA^R^ colonies per mg of dry tissue. The experiments were repeated three times.

### 2.4. Mutant Lines

The introduction of mutated codons into the genome via knock-in was realized using homology-directed repair, initiated by the induction of DSB within the *PpSMC6* locus (Pp3c11_11190) with Cas9 nuclease. Two double-stranded DNA donor templates, each consisting of 57 base pairs, were used for the induction of Glycine (G) to Arginine (R) mutations at 514 or 517 amino acid positions, respectively. These donor templates were designed to encompass the conserved hinge PPxGPxG motive of the *PpSMC6*, and synthesized as complementary oligonucleotide pairs (1594 and 1595 for *G514R*, and 1461 and 1462 for *G517R* mutation, Table S1).

Template oligos of the *G514R* mutation contained a +2547 GGA to CGC substitution and two silent mutations, specifically a C2543A and T2546C, which disrupted a BsmBI cleavage site. Similar templates were designed to generate the *G517R* mutation by +2556 GGA to CGC replacement.

The Cas9/sgRNA expression vectors were generated through the Gateway LR reaction, involving the recombination of the entry vector with the *PpSMC6* sgRNA spacer and the destination vector. The gateway destination vector pMK-Cas9-gate (Addgene plasmid #113743) with the Cas9 expression and NPTII selection cassette was used along with the entry vector pENTR-PpU6sgRNA-L1L2 (Addgene plasmid #113735), harboring the PpU6 promoter and the sgRNA scaffold [20]. A PpSMC6-specific sgRNA spacer was synthesized as a pair of complementary oligonucleotides (1459 and 1460; Table S1).

These DNA constructs, together with their respective donor templates, the annealed oligos 1594 and pKA1595 for *G514R* and 1461 and 1462 for the *G517R* mutation, were co-transformed into protoplasts via PEG-mediated transformation.

After a five-day regeneration period, the transformed protoplasts were transferred to a BCDAT medium supplemented with 50 mg/L G418 for selection. Following a one-week selection, the G418-resistant colonies were propagated. Crude extracts from the young tissues of these lines were employed for the PCR amplification of the genomic DNA encompassing the edited sites, using primers 711 + 839. The resulting PCR products were digested with BsmBI and electrophoresed. Three *Ppsmc6_G514R*#6, 20, 28 and three *Ppsmc6_G517R*#2, 3, 4 lines providing uncleaved PCR product were further analyzed and *SMC6* transcript sequenced for the accurate introduction of mutation. The lines *Ppsmc6_G514R#20* and *Ppsmc6_G517R#3* (Figure S1) were picked, denominated as *Ppsmc6_G514R* and *Ppsmc6_G517R*, respectively, and used in this study.

The line *Ppsmc6_dCas* (formerly *Ppsmc6-3*), with the sgRNA-directed immobilization of ‘dead’ Cas (dCas) nuclease to the hinge, leading to the attenuated transcription of *SMC6,* has been described earlier [13].

### 2.5. Yeast Two-Hybrid (Y2H) Constructs and Assays

The classical Gal4-based Y2H system was used to analyze protein–protein interactions as described previously [11,12,21]. Briefly, *PpSMC6_G514R*, *PpSMC6_G517R* (Pp3c11_11190V3.1), *PpSMC5* (Pp3c24_4940V3.1), *PpNSE5* (Pp3c13_1090V3.1) and *PpNSE6* (Pp3c4_7040V3.1) genes were PCR-amplified from cDNA and subcloned into pGBKT7- or pGADT7-based plasmids, which were co-transformed into the *S. cerevisiae* PJ69-4a strain and selected on SD -Leu/-Trp plates. Drop tests were carried out on SD-Leu/-Trp/-His plates at 28 °C. Each combination was transformed at least three times with three independent drop test replicas.

### 2.6. DNA Isolation and Analysis of rDNA Copy Numbers

DNA was isolated from a seven-day-old protonema culture, according to [22]. The DNA quality was checked by electrophoresis in a 1% (*w*/*v*) agarose gel stained with ethidium bromide, and concentration was determined using Gene Ruler 1 kb DNA Ladder (Thermo Scientific, Waltham, MA, USA) as a standard. qPCR for the estimation of rDNA copy number was performed in triplicates for three biological samples to analyze 18S rDNA (primer combination Pp18S_A and Pp18S_B) and 5S rDNA (primer combination Pp_5S_F and Pp_5S_R) and normalized to ubiquitin used as a reference gene (primer combination ubqFw and ubqRev [15], Table S1), using qPCRBIO SyGreen Mix Lo-ROX (PCR Biosystems, London, UK) in Stratagene-MX3005P Quantitative PCR System (La Jolla, CA, USA). The data were statistically analyzed using the two-tailed unpaired Student’s *t*-test.

### 2.7. Morphotype Analysis

After 1 month of growth of WT and mutant lines on the BCDAT medium, pictures of whole colonies were taken using a Canon EOS77D camera with a Canon EF 28–135 mm f/3.5–5.6 lens. The details of the gametophores, antheridia and archegonia were photographed using a Stereomicroscope Leica M205FA and a Plan-Apochromat 2.0× lens.

### 2.8. Microscopic Analysis

Ten-day-old fibers of protonema were stained with 10 µg/mL propidium iodide (PI) (Sigma-Aldrich, Schnelldorf, Germany) in liquid BCDAT medium, mounted onto a glass slide and analyzed by a Spinning disk (SD) microscope Nikon Eclipse Ti-E, inverted (Nikon, Minato, Japan) with a Yokogawa CSU-W1 SD unit (50 mm), on a Nikon Ti-E platform, Laser box MLC400 (Agilent. Santa Clara, CA, USA), Zyla cMOS camera (Andor, Belfast, Ireland), and a Nikon Plan-Apochromat L 10×/0.45 lens.

### 2.9. Single-Cell Gel Electrophoresis (Comet) Assay

Repair kinetics were estimated in 1 d and 7 d protonema cultures after bleomycin treatment. Tissue was either flash frozen in liquid N_2_ (repair t = 0) or left to recover in the liquid BCDAT medium for the indicated repair times, and frozen afterwards. DSBs induced by bleomycin treatment were detected by a ‘comet’ assay in neutral conditions [16,17]. Comets with a ‘tail’ of damaged DNA pulled out of the nucleus by an electric field were stained with SYBR Gold dye (Molecular Probes/Invitrogen, Eugene, OR, USA), observed in epifluorescence with a Nikon Eclipse 800 microscope and evaluated with LUCIA Comet cytogenetic software (LIM Inc., Prague, Czech Republic). The fraction of DNA in comet tails (% tail-DNA) was used as a measure of DNA damage. In each experiment, the % tail-DNA was measured at seven time points: 0, 3, 5, 10, 20, 60 and 180 min after the treatment, and in control tissue without a treatment. Measurements obtained in three independent experiments with four technical replicates (individually evaluated comet slides) totaled in at least 300 comets analyzed per experimental point. Data plotted as % of remaining damage were analyzed in the Prism10 (GraphPad Software Inc., San Diego, CA, USA) program for each line.

### 2.10. GT Assay

GT efficiencies were assayed by transforming *P. patens* protoplasts with the *APT*-based targeting construct [23]. Linear pKA255 targeting vector with hygromycin-resistance cassette (Hygr^R^) flanked by genomic *PpAPT* derived sequences (Figure 8a) was PCR-amplified and delivered by PEG-mediated transformation to protoplasts of WT and mutated lines [24]. The transformed protoplasts of each line were spread on four Petri plates with the BCDAT medium overlaid with a cellophane disk. Regenerating protoplasts were counted after 5 days and then transferred on cellophane to new Petri dishes with BCDAT medium with 30 mg/L hygromycin for a week for cultivation, after which Hyg^R^ colonies appeared. The relative transformation index (RTF) was calculated as the ratio of Hygr^R^ transformants to the total number of regenerating protoplasts. Cellophane disks with Hygr^R^ transformants were transferred onto new plates with BCDAT medium containing 5 μM 2FA for the detection of GT events in *APT*. After 3 weeks, 2FA^R^ colonies were counted and the frequency of GT events was expressed as a ratio of 2FA^R^ vs Hygr^R^ colonies. The experiment was repeated three times and statistically analyzed using the Fisher exact test.

## 3. Results

### 3.1. Generation and Analysis of the P. patens SMC Hinge Mutants

**Figure 1:**
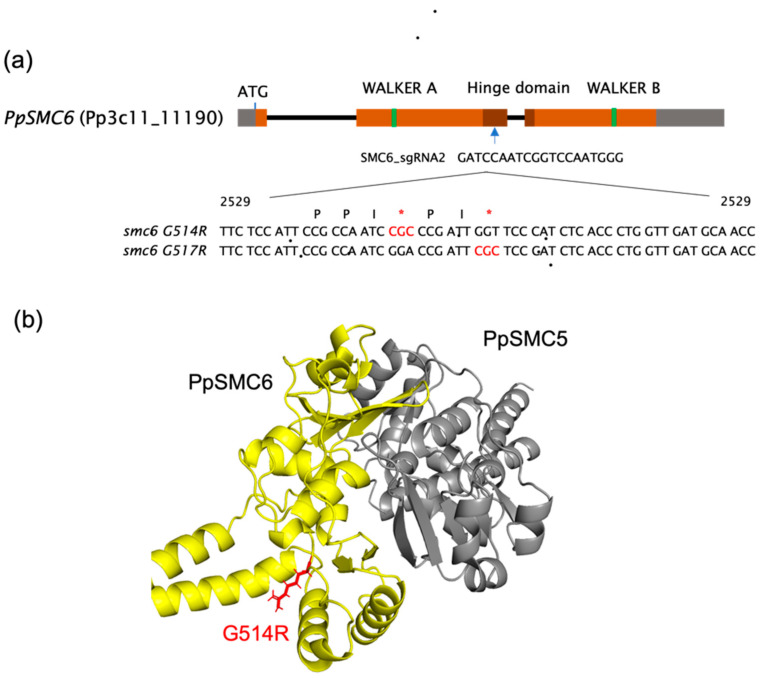
(**a**) A map of *P. patens SMC6* locus (Pp3c11_11190) representing the structure of the SMC6 protein and the localization of the hinge (dark brown). Indicated is the site of sgRNA annealing, which directs either binding of nonfunctional ‘dead’ Cas9 nuclease (dCas9) for the attenuation of *SMC6* transcription in *Ppsmc6_dCas* line [13], or CRISPR/Cas9 cut to help the recombinational introduction of the donor templates. Within the context of the donor template oligonucleotides, the localization of the conserved PPxGPxG motif is shown [10] with red asterisks highlighting point mutations (*G514R* and *G517R*). The positions of nucleotides are relative to the SMC6 ATG start codon, where A has position number 1. (**b**) A molecular cartoon depiction of the *P. patens* SMC5/6 heterodimeric hinge with subdomains of SMC6 (yellow) with red arginine (R514) residue tail and SMC5 (gray).

Using CRISPR/Cas9-directed oligonucleotide replacement of the original gene sequence, we introduced the *G514R* mutation and the close *G517R* mutation to the hinge of the *SMC6* gene in the genome of *P. patens* (Figure 1a,b) and obtained viable *Ppsmc6_G514R* and *Ppsmc6_G517R* lines, which both showed exclusive transcription of only the mutated gene for the respective line.

Although all the lines were viable, the fresh weight of growing explant colonies slightly differed. The average weight of the untreated colony after three weeks of growth from all experiments was 13.25 mg for WT, 10.95 mg for *Ppsmc6_dCas*, 9.55 mg for *Ppsmc6_G514R* and 13.55 mg for the *Ppsmc6_G517R* line.

**Figure 2:**
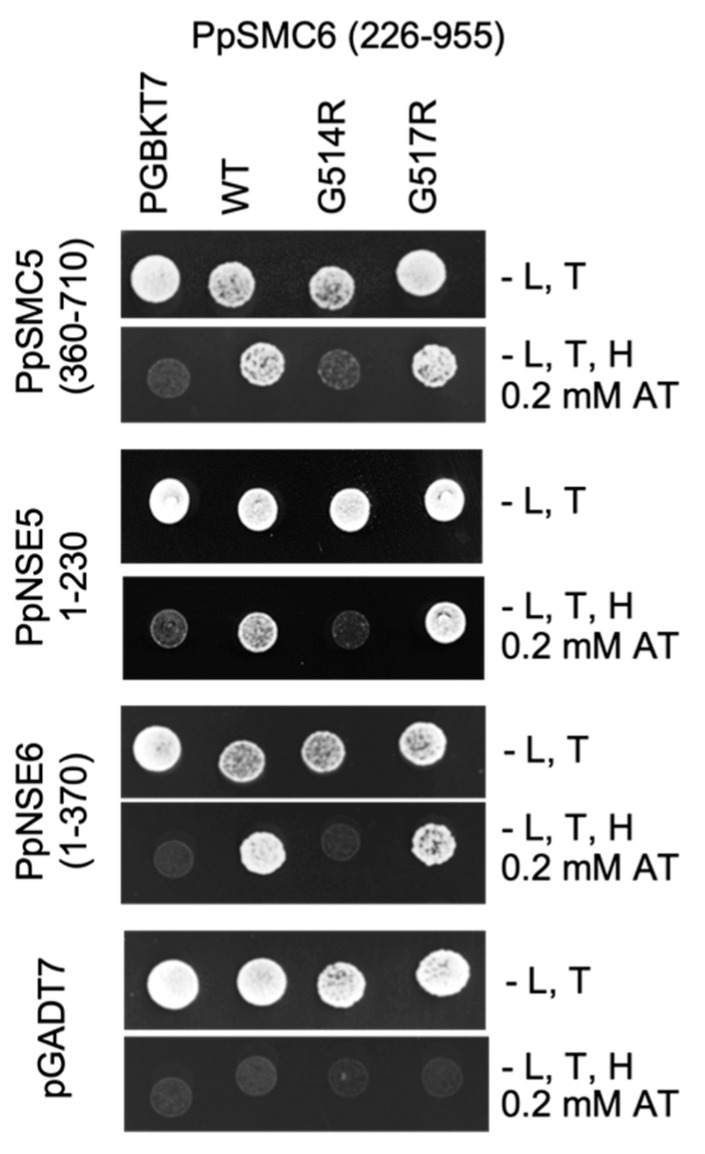
Detailed mapping of WT and mutant PpSMC6 binding to PpSMC5, PpNSE5 and PpNSE6 by Y2H. Interacting parts are indicated in the image. While the *G514R* point mutation interrupts the interaction of PpSMC6 with PpSMC5, PpNSE5 and PpNSE6, the *G517R* mutation has no effect, and interactions remain the same as with WT.

To analyze the impact of the *G514R* and *G517R* mutations on the interactions of PpSMC6 with PpSMC5, PpNSE5, and PpNSE6, we used the Y2H assay [11,12]. A PpSMC6(aa226-955) fragment, containing arm and hinge domains, was tested against the PpSMC5 hinge-containing fragment, and PpNSE5 and PpNSE6 constructs, which were already verified in our previous work [11,12]. The *G514R* mutation disrupts the direct interaction of PpSMC6 not only with PpSMC5, but also with PpNSE5 and PpNSE6 (Figure 2 and Supplementary Figure S2). It seems likely that the *G514R* mutation has a serious structural consequence which does not specifically disrupt a single, but rather several PpSMC6 interactions. Surprisingly, the nearby *G517R* mutation had no effect, and the interplay among the proteins of the SMC5/6 complex remains the same as in WT (Figure 2).

### 3.2. Morphology of P. patens SMC6-Hinge Mutants

**Figure 3:**
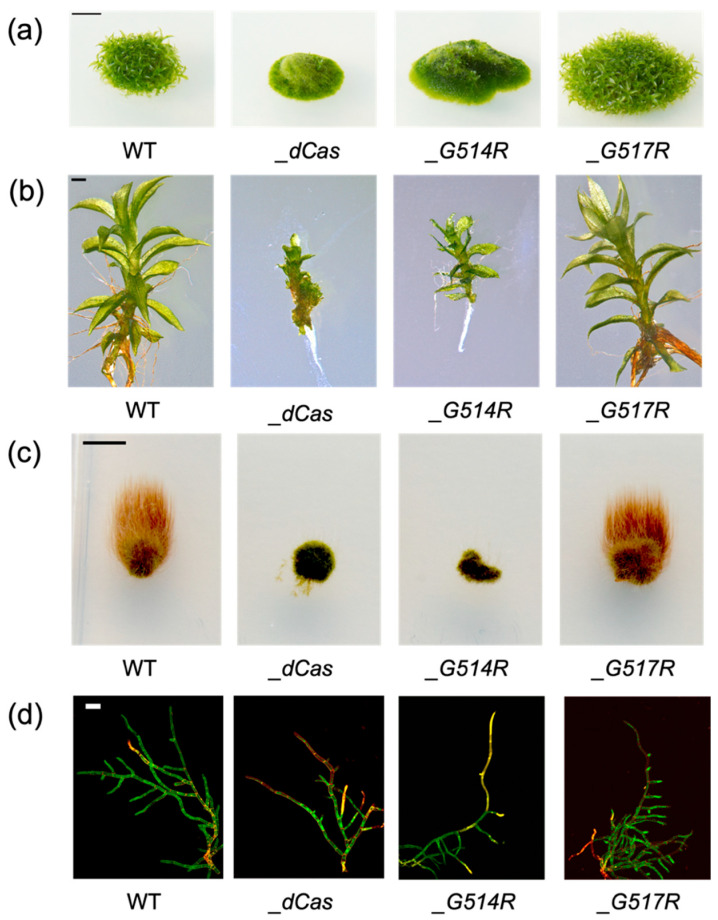
(**a**) Morphology of *P. patens* WT and *Ppsmc6_dCas* (*_dCas*), *Ppsmc6_G514R* (*_G514R*) and *Ppsmc6_G517R* (*_G517R*) mutant lines of 1-month-old colonies grown on BCDAT medium. Scale bar 5 mm. (**b**) Close-up view of 1-month-old gametophores. Scale bar 1 mm. (**c**) Caulonema growth in the dark for three weeks on BCDAT medium with 0.5% sucrose. Scale bar 5 mm. (**d**) PI stained 10-day-old protonema fibers grown on BCDAT medium. Scale bar 100 µm.

Both hinge mutations are not toxic to the extent that they allow growth and viability of the established *Ppsmc6_G514R* and *Ppsmc6_G517R* lines (Figure 3a). The *Ppsmc6_G514R* line has a developmentally strongly aberrant morphology similar to the *Ppsmc6_dCas* line and reduced weight gain by approximately 10 to 15%. Gametophore development is dramatically reduced in *Ppsmc6_G514R* plants and almost completely inhibited in *Ppsmc6_dCas* (Figure 3a), with rarely occurring strongly aberrant gametophores (Figure 3b). The growth of juvenile gametophores is also completely arrested (Figure 3c). Apical cells of *Ppsmc6_dCas* and *Ppsmc6_G514R* do grow; however, protonema fibers appear aberrant and failing to develop gametophores. Branching in *Ppsmc6_dCas* is almost completely abolished by the death of branching cells (Figure 3d). In contrast, the viability and development of gametophores and juvenile gametophores of *Ppsmc6_G517R* seem to be unaffected at all. In general, the morphology of the *Ppsmc6_G517R* line is more or less similar to WT.

### 3.3. Growth-Response of P. patens SMC6-Hinge Mutants to Bleomycin

To characterize the response of *PpSMC6* mutants to DNA damage, we compared their growth responses to multiple types of lesions, such as DNA breaks, particularly DSBs, abasic sites and various kinds of oxidative damage induced by the radiomimetic drug bleomycin. These lesions represent blocks for DNA replication and are removed, repaired or bypassed by various error-free as well as error-prone pathways. The sensitivity was assayed as the ability of tissue to recover from bleomycin treatment during the following 3 weeks of recovery.

**Figure 4:**
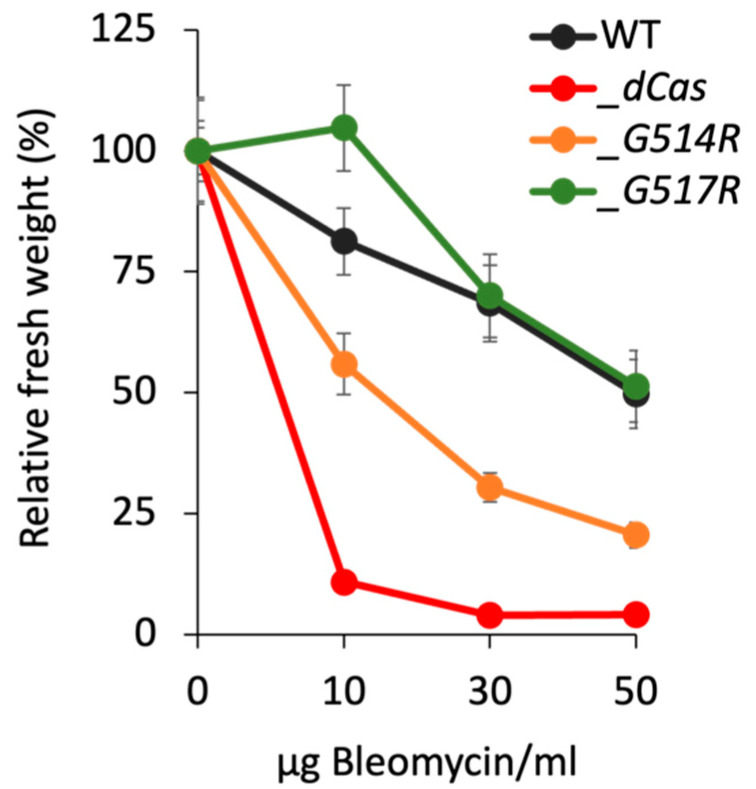
Growth responses of *P. patens* WT (black) and *Ppsmc6_dCas* (*_dCas*) (red), *Ppsmc6_G514R* (*_G514R*) (light brown) and *Ppsmc6_G517R* (*_G517R*) (green) mutant lines to 1 h treatment with 10, 30 and 50 µg/mL of bleomycin. After the treatment, the explants were incubated on a drug-free BCDAT medium under standard growth conditions for 3 weeks. Then, for each experimental point, the weight of treated plants collected from two replica plates was normalized to the weight of untreated plants and plotted as relative fresh weight, which was set by default to 100. Error bars indicate SE.

The *Ppsmc6_dCas* line (*p* < 0.01) did not survive exposure even to the lowest tested concentration of bleomycin at 10 µg/mL, while the *Ppsmc6_G514R* line (*p* < 0.05) remained partially viable even after treatment with bleomycin at a dose of 50 µg/mL. Contrary to *G514R*, the *G517R* mutation had a significantly different effect (*p* < 0.05) on the survival of bleomycin treatment, particularly at a lower bleomycin concentration (10 µg/mL), manifested as stimulation of the growth of *Ppsmc6_G517R* explants even over WT (Figure 4).

### 3.4. Spontaneous and Induced Mutagenesis in P. patens SMC6-Hinge Mutants

The frequency of spontaneous or bleomycin-induced 2FA^R^ colonies in a population of 1 d regenerating protonema fragments was used as a marker to assess the mutator phenotype at the *PpAPT* locus. Since spontaneous APT inactivation is likely to arise from replication-associated errors rather than any other error-prone DNA repair, we also tested mutagenesis induced by exposure to a low concentration of bleomycin, which generates a broad spectrum of DNA damage.

**Figure 5:**
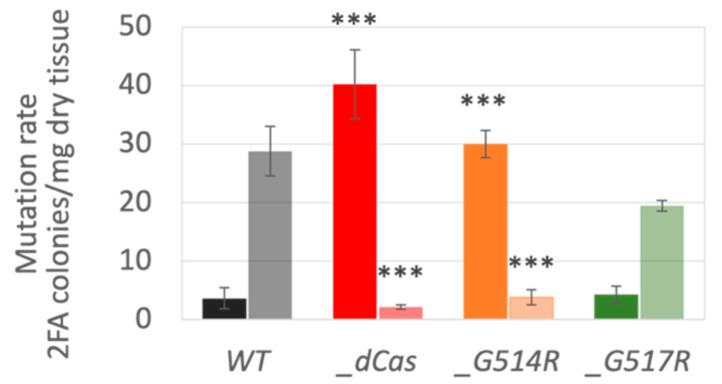
Mutagenesis in the *APT* locus of *P. patens* WT (black) and *Ppsmc6_dCas* (*_dCas*) (red), *Ppsmc6_G514R* (*_G514R*) (light brown) and *Ppsmc6_G517R* (*_G517R*) (green) mutant lines. Inactivation of *APT* by mutation leads to resistance to 2FA [19]. The 2FA surviving colonies were counted and expressed as the number of 2FA^R^ colonies per mg of dry tissue. Spontaneous mutations are depicted as dark, 100% full-colored columns, while bleomycin-induced mutations are represented as half, 50% colored columns. Student’s *t*-test: *** *p* < 0.001, and error bars represent SE.

Compared with the WT and *Ppsmc6_G517R* lines, *Ppsmc6_dCas* and *Ppsmc6_G514R* displayed a strong spontaneous mutator phenotype (Figure 5). Mutations were increased to 40 and 30 colonies/mg dry tissue in the *Ppsmc6_dCas* and *Ppsmc6_G514R* lines, respectively, compared to only 4 and 5 colonies/mg dry tissue in WT and *Ppsmc6_G517R*. These mutations are most likely the result of a replication or bypass errors that inactivated APT. In contrast to spontaneous mutagenesis, introduction of DNA lesions by bleomycin pretreatment induced error-prone repair-related mutagenesis only in the WT and *Ppsmc6_G517R* lines (28 and 19 colonies/mg dry tissue), whereas in the *Ppsmc6_dCas* and *Ppsmc6_G514R* lines, any *APT*-related mutagenesis was virtually eliminated.

### 3.5. DSB Repair in P. patens SMC6-Hinge Mutants Is Not Reduced

The time course of double-strand break (DSB) repair followed by the comet assay in *P. patens* WT and *PpSMC6*-hinge mutants best fits the kinetics of “biphasic decay” as determined by data analysis using the Prism/GraphPad program. During the first, fast phase, DSBs are mostly directly removed by the error-prone NHEJ mechanism, while during the slow phase, DSBs are preferentially repaired by recombination [25].

**Figure 6:**
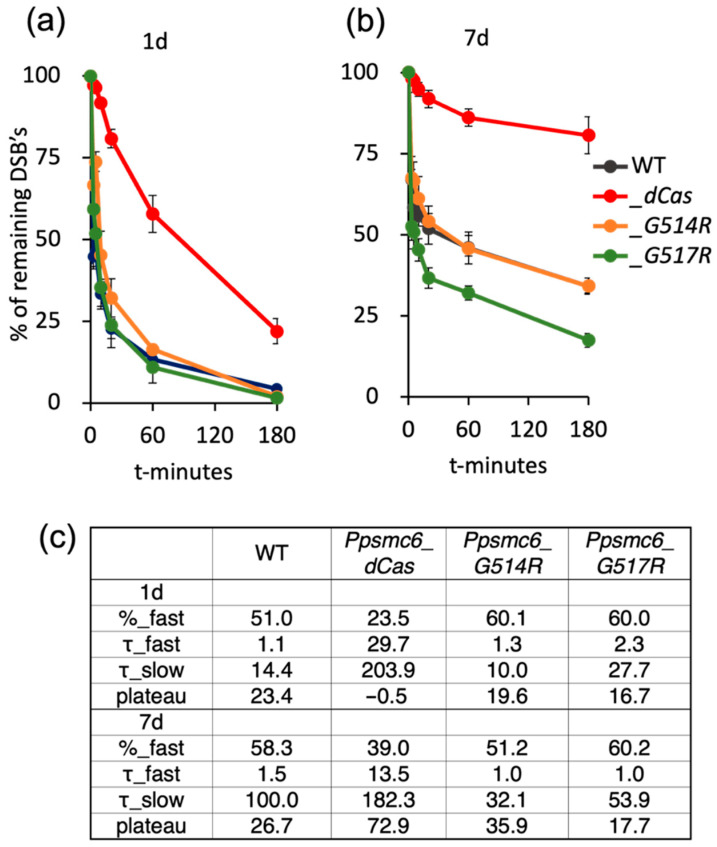
DSB repair kinetics determined by ‘comet’ assay in 1 d (**a**) and 7 d (**b**) tissue of *P. patens* WT (black) and *Ppsmc6_dCas* (*_dCas*) (red), *Ppsmc6_G514R* (*_G514R*) (light brown) and *Ppsmc6_G517R* (*_G517R*) (green) mutant lines. Protonemata were treated with 30 μg bleomycin/mL for 1 h, and repair kinetics was measured as % of remaining DSBs after 0, 3, 5, 10, 20, 60 and 180 min of repair recovery. Maximum damage was normalized as 100% at t = 0 for all lines. Error bars indicate SE. Analysis of DSB time course data with the Prism/GraphPad program returned the best fit to ‘Two phase decay’ kinetic. Table (**c**) lists kinetic parameters: Fraction of fast phase (%-fast), half-lives (τ) during fast and slow phase, and plateau representing remaining damage.

The hinge mutations, *G514R* and *G517R*, have no effect on the DSB repair in dividing, 1d cells (Figure 6a), but the *Ppsmc6_dCas* line shows a strong reduction (*p* < 0.01) in repair and the absence of a rapid repair phase (Figure 6a,c). Also, in differentiated, 7 d cells, the line *Ppsmc6_dCas* has a reduced repair rate of DSBs (*p* < 0.01) due to the lack of its rapid fraction, while *Ppsmc6_G514R* and WTs repaired rapidly and at the same pace more than 50% of DSBs in 1 h. In *Ppsmc6_G517R,* more than 50% of DSBs were repaired in 10 min, significantly faster than in WT (*p* < 0.01) or *Ppsmc6_G514R* (*p* < 0.01), due to the highest fraction of rapid repair, a repair rate that was twice as fast during the slow phase, and the lowest plateau of remaining damage (Figure 6b,c). The true fast DSB repair in *Ppsmc6_G517R* was confirmed by ruling out false rapid repair caused by the formation of DNA–protein cross-links by treating comet slides with Proteinase K.

### 3.6. The Role of P. patens SMC6-Hinge Mutants in Maintenance of Genome Stability

**Figure 7:**
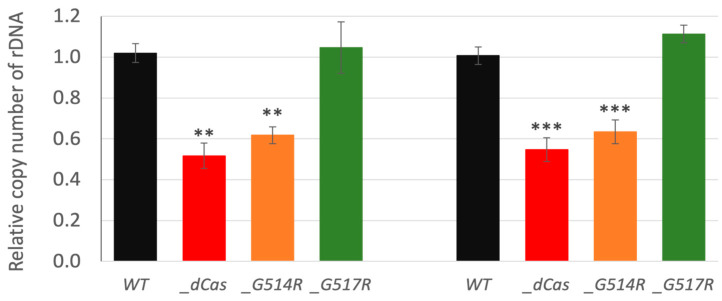
Relative copy number of 18S and 5S rDNA were measured by qPCR in *P. patens* WT (black), by default set to 1, and mutant lines *Ppsmc6_dCas* (*_dCas*) (red), *Ppsmc6_G514R* (*_G514R*) (light brown) and *Ppsmc6_G517R* (*_G517R*) (green). Numbers of rDNA copies in *Ppsmc6_dCas* and *Ppsmc6_G514R* are significantly reduced in comparison to WT and *Ppsmc6_G517R*. Student’s *t*-test: ** *p* < 0.01; *** *p* < 0.001, and error bars represent SE.

The SMC5/6 complex is actively involved in overcoming chromosome sites that are difficult to replicate. This is well documented by its role at replication fork barrier sites in the ribosomal DNA (rDNA) array of yeast [26]. We followed the influence of hinge mutations on the rDNA maintenance by measuring changes in the number of copies within arrays of 18S and 5S rDNA (Figure 7). Levels of rDNA copies were significantly reduced in *Ppsmc6_dCas* and *Ppsmc6_G514R* to 50% and 60%, respectively, whilst line *Ppsmc6_G517R* remained similar to WT.

### 3.7. Absence of GT in P. patens SMC6 G514R Mutant

**Figure 8:**
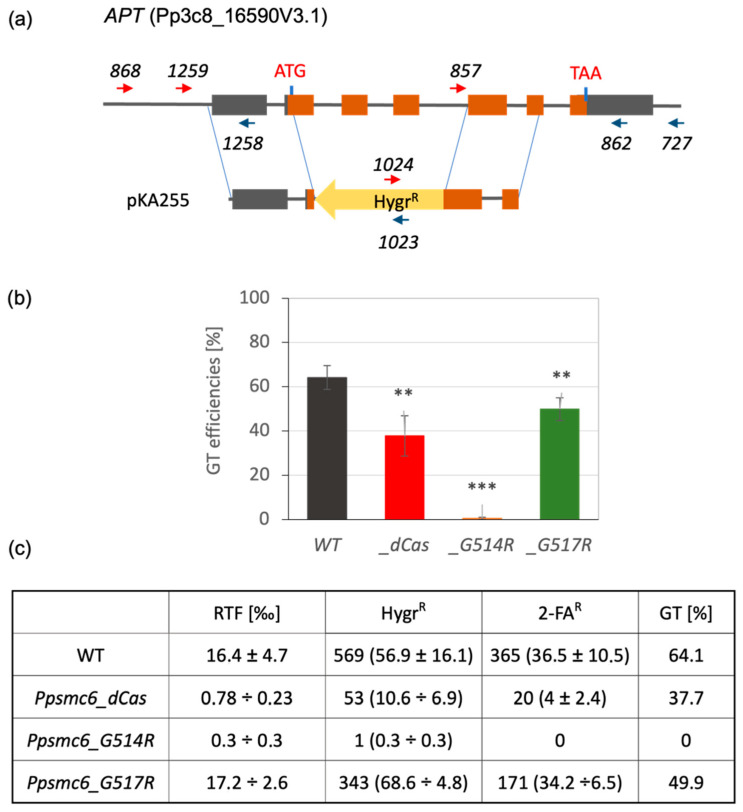
GT assay [23]. (**a**) Schematic drawing of *P. patens APT* locus and of targeting construct pKA255. The boxes represent exons, and the gray shading indicates UTR regions. Arrows mark the position of primers used to genotype and sequence the plants by PCR [13]. (**b**) GT efficiencies in *Ppsmc6_dCas* (*_dCas*) (red), *Ppsmc6_G514R* (*_G514R*) (light brown) and *Ppsmc6_G517R* (*_G517R*) (green) mutant lines in comparison to *P. patens* WT (black). Student’s *t*-test: ** *p* < 0.01; *** *p* < 0.001, and error bars represent SE. (**c**) Table of numerical values of transformation and of GT efficiencies. Relative transformation index RTF [in ‰] is the frequency of Hygr^R^ transgenic clones in the whole regenerating protoplast population; Hygr^R^ and 2FA^R^ indicate total and average ± SE resistant clones determined in three independent experiments. Efficiency of GT [in %] expresses the frequency of 2FA^R^ resistant clones among the population of Hygr^R^ transgenic clones.

Transformation frequencies were determined at every stage of selection, and the final GT efficiencies were expressed as the frequency of 2FA^R^ clones among Hygr^R^-resistant transgenic transformants. The targeting vector (Figure 8a) disrupts the *PpAPT* gene across the previously identified mutation hotspot in the second exon site [13,27] and provides transformants with resistance to hygromycin. The initial efficiency of transformation depends on the genotype and decreases in the range from 17.2 × 10^−3^ for *Ppsmc6_G517R*, 16.4 × 10^−3^ for WT, 0.78 × 10^−3^ for *Ppsmc6_dCas,* to just 0.3 × 10^−3^ for a single transformant of *Ppsmc6_G514R*. The gene-targeting efficiency, calculated as the ratio of 2FA^R^ clones to primary Hygr^R^ transformants, also depends on the genotype and decreases from 64.1% for WT to 49.9% for *Ppsmc6_G517R* and 37.7% for *Ppsmc6_dCas*, respectively, while in *Ppsmc6_G514R,* where no 2FA^R^ clone was recovered gene-targeting is completely abolished (Figure 8c). A PCR analysis of randomly selected 2FA^R^ transformants of each genotype was performed to assess the accuracy of integration. It revealed that in two 2FA^R^ transformants of each line, except for *Ppsmc6_G514R,* the insert borders had accurate insertion without mutations at either end. This experiment showed, firstly, that SMC6 is involved in transformation, and, secondly, that it plays an important role during targeted insertion. If we assume targeted integration as closely connected with HR, our results also show that intact SMC6 directly participates in the process of HR.

## 4. Discussion

In this study, we characterized the effects of mutations in the hinge region of SMC6 on the activities of the SMC5/6 complex and on the stability and repair of genomic DNA in the moss *P. patens*. Regardless of *SMC6* being essential gene in *P. patens,* we were able to introduce two nearby G to R mutations at aa514 and aa517 in the conserved motif ‘PPxGPxG’ of the hinge domain, and recovered the viable lines *Ppsmc6_G514R* and *Ppsmc6_G517R*, though very different in morphology and phenotype. 

The line with the *G514R* mutation had an aberrant morphology and altered phenotype, which, in most features, paralleled the previously constructed *Ppsmc6_dCas* line [13]. Changes due to the *G514R* mutation may be a consequence of disrupted interactions within the PpSMC5/6 complex (Figure 2). Besides the expected hinge-hinge interaction with PpSMC5, we recently described the interaction of an arm fragment of PpSMC6 with the CANIN domain (aa1–370) of the PpNSE6 protein [11] and PpNSE5 [12]. These interactions are lost due to the *G514R* mutation, suggesting the severe loss of structure and functions of the SMC6 protein. Consistent with the structural defect of the PpSMC6/G514R protein, the viability and genome stability of the *Ppsmc6_G514R* line were severely compromised. Similarly, the yeast *S. pombe* SpSMC6/G551R mutant cells were viable but exhibited severe temperature sensitivity and genome stability phenotypes [10]. Interestingly, most of the phenotypic features, such as morphology (Figure 3), spontaneous mutagenesis (Figure 5) and maintenance of rDNA arrays (Figure 7) of *Ppsmc6_G514R*, resemble those of the *PpNSE5KO* and *PpNSE6KO* mutants [11,12]; therefore, it is tempting to hypothesize that when the control of *PpNSE5* and *PpNSE6* through their interactions is lost, the mutated phenotype is manifested.

Surprisingly, a nearby *G517R* mutation of the same conserved ‘PPxGPxG’ motif had no effect on the interactions within the SMC5/6 complex. In some aspects, such as resistance to DNA damage and DSB repair kinetics, it even manifests a more ‘viable’ phenotype than WT, suggesting that hinges may play some regulatory role. Currently, it is difficult to interpret the antagonistic behavior of the *G517R* mutation, because its effect on the above protein–protein interactions is minimal when compared to the ‘catastrophic’ havoc introduced by the *G514R* mutation. However, based on experimental data, the effect of the *G517R* mutation can be considered as a stabilizing and/or supporting factor for the functions of SMC5/6 hinges.

Lines with the hinge mutations *G514R* and *G517R* differ in the survival and maintenance of genome stability. In the *Ppsmc6_G514R* line, we observed spontaneous and induced mutagenesis and rDNA copy numbers very similar to those in the *Ppsmc6_dCas* line, a line with reduced WT *SMC6* transcription. However, the two lines differ in their sensitivity to bleomycin, with 50% of *Ppsmc6_G514R* explants surviving bleomycin treatment at a concentration of 10 µg/mL, a concentration at which *Ppsmc6_dCas* explants are virtually dead. In the *Ppsmc6_G517R* line, the hinge mutation had virtually no effect and its responses were the same as WT, with the exception of increased survival after exposure to a low concentration of bleomycin (10 µg/mL) compared to WT.

The main differences between *smc6* mutants were observed during DSB repair. Surprisingly, no reduction in the DSB repair rate was observed in lines with the *G514R* and *G517R* point mutations compared to WT. Moreover, the *Ppsmc6_G517R* line repairs DSBs faster than WT, due to an increased repair rate during the second, slower phase of repair. The increased rate also leads to higher repair efficiency and less residual damage (plateau in Figure 6c). In contrast, the *Ppsmc6_dCas* line shows severe defects of DSB repair in both dividing, 1 d and differentiated, 7 d cells, suggesting that a sufficient level of SMC6 is critical for acute fast DNA repair, while SMC6 interactions are not. Notably, plant viability, rDNA stability and spontaneous mutagenesis were compromised by both the reduced levels of SMC6 and the lack of its interactions.

The roles of both mutations can also be illustrated in the process of GT at the *APT* locus (GT), which strongly depends on the homologous recombination. We can sort the efficiency of GT in *SMC6* mutant lines (Figure 8) into three groups. The highest efficiency of GT was achieved in WT (64.1%), moderate in *Ppsmc6_G517R* (49.9%) and *Ppsmc6_dCas* (37.7%) lines, and in line *Ppsmc6_G514R*, GT was completely absent. To interpret these results, it is necessary to recognize the key role of SMC6 interactions in WT, with mutated lines *Ppsmc6_dCas* (WT with attenuated transcription) and *Ppsmc6_G517R* (Figure 2) on the one hand, and their loss in *Ppsmc6_G514R* on the other. It should be noted that the *Ppsmc6_G514R* line exclusively transcribes the mutated SMC6 gene with G514R (Figure S1), and this mutation is solely responsible for preventing GT, while reduced transcription of *SMC6* attenuates, but does not prevent GT. Clearly, the lack of interactions is harmful to GT, whereas reduced levels of SMC6 are not. Together, reduced levels of SMC6 and the lack of its interactions have similar severe consequences for plant viability, rDNA stability and spontaneous mutagenesis. However, SMC6 levels affect more the acute fast DNA repair processes, while the disturbed interaction in *G514R* mutant affects different processes, like GT.

## 5. Conclusions

Based on the results, we conclude that in *P. patens* moss, a sufficient amount of SMC6 and its unperturbed interactions with SMC5, NSE5 and NSE6 are essential for normal development and genome stability, such as low spontaneous mutagenesis, active double-strand breaks (DSB) repair and genomic rDNA maintenance. Acute DSB repair is not affected by disrupted interactions in *Ppsmc6_G514R*. However, the loss of contacts between the SMC6 and SMC5, NSE5 and NSE6 fully prevents GT, in contrast to only a modest effect on GT efficiency in the *Ppsmc6_dCas* line with reduced SMC6 expression. Therefore, we propose that the loss of interactions with the above factors has different impact than reduced levels of fully functional SMC6 proteins, particularly in acute (NHEJ) and slow (HR) repair.

## Data Availability

The original contributions presented in this study are included in the article/Supplementary Materials. Further inquiries can be directed to the corresponding authors.

## Supplementary Materials

The following supporting information can be downloaded at: https://www.mdpi.com/article/10.3390/genes16091091/s1, Table S1: Used oligonucleotides, Figure S1: RT-PCR transcripts of SMC6 in Ppsmc6_G514R and Ppsmc6_G517R lines, Figure S2: Analysis of the PpSMC6 interactions [28,29].

## Abbreviations

The following abbreviations are used in this manuscript:

DSB	DNA double strand break
dsDNA	double-stranded DNA
2FA	2-Fluoroadenin
GT	Gene Targeting
HR	Homologous Recombination
NHEJ	Non-Homologous End Joining (DSB repair pathway)
NPTII	Neomycin PhosphoTransferase conferring resistance to Kanamycin/G418
PI	Propidium iodide
ssDNA	single-stranded DNA
τ	Half-life
Y2H	Yeast two Hybrid system
