# Supplementary material for: The Phenotype of Physcomitrium patens SMC6 Mutant with Interrupted Hinge Interactions
Source: Genes (Basel). 2025 Sep 16;16(9):1091. doi: 10.3390/genes16091091 (PMC12469541; doi:10.3390/genes16091091)
Supplement: Supplementary file 1 [file genes-16-01091-s001.zip › Supplement Figures S1, 2.pdf]

Figure S1: RT-PCR transcripts of *SMC6* in *Ppsmc6_G514R* and *Ppsmc6_G517R* lines.

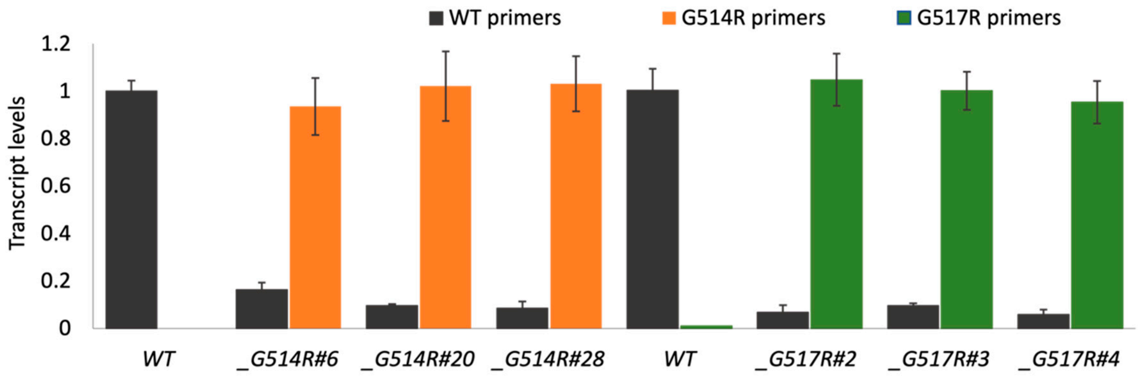

Figure S1. Relative *SMC6* transcript levels measured by qPCR in *P. patens* WT, by default set to 1, and mutant lines *Ppsmc6_G514R#6,20,28* (*_G514R#6,20,28*) and *Ppsmc6_G517R#2,3,4* (*_G517R#2,3,4*). From all lines analyzed, the *Ppsmc6_G514R#20* and *Ppsmc6_G517R#3* were picked, denominated as *Ppsmc6_G514R* and *Ppsmc6_G517R*, respectively and used in this study. Primers used are listed in Supplementary Table S1. Error bars represent SE.

Figure S2: Analysis of the PpSMC6 interactions.

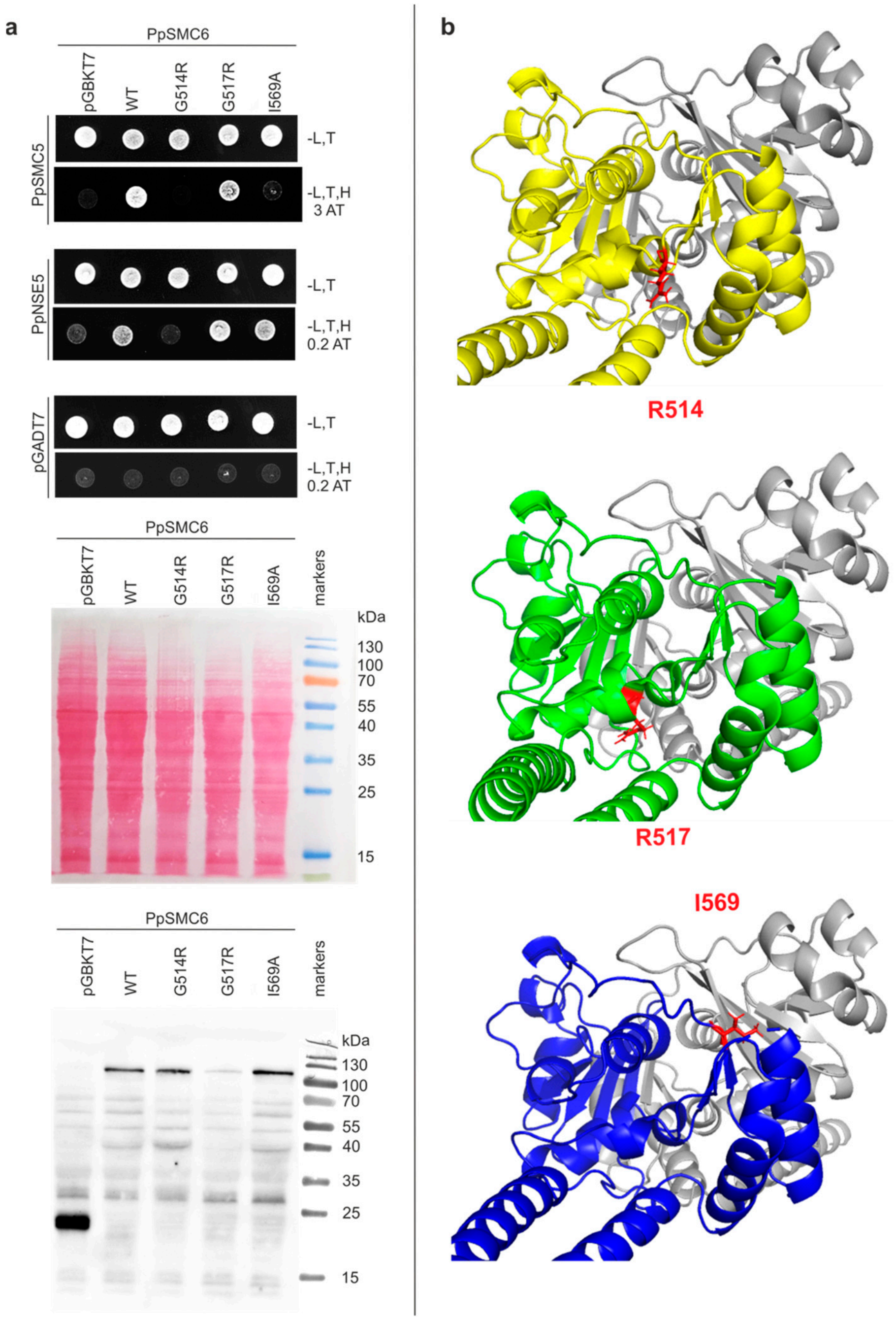

Figure S2. Detailed mapping of the PpSMC6 WT and mutants. **(a)** The G517R mutation has no effect on PpSMC6 interactions, while G514R interrupts the interaction with both PpSMC5 and PpNSE5, suggesting severe structural defects. The I569A mutation specifically disturbs only binding to PpSMC5 as it affects only the hinge-hinge binding surface. Interacting part of each protein is indicated in the picture. The Y2H protein-protein interactions were scored by the growth of the yeast PJ69-4 transformants on the plates without Leu, Trp, His (-L,T,H), and with the indicated concentration of 3-Amino-1,2,4-triazole (AT). Control plates were lacking only Leu and Trp (-L,T). Empty pGBKT7 and pGADT7 vectors were used as negative controls. Expression of the Gal4BD-PpSMC6 constructs in yeast PJ69-4 strain was verified using the anti-Myc-HRP antibody (bottom panel). The middle panel shows an equal loading of protein extracts stained with Ponceau S red. **(b)** Molecular-cartoon depiction of the *P. paten*s SMC5/6 heterodimeric hinge with subdomains of PpSMC6 (colored) and PpSMC5 (grey) with red-labeled position of the concerned amino acid.

Supporting methods

Most methods are described in the main paper.

Protein expression analysis

Yeast PJ69-4 cells were grown in YPD to OD_{600} ~ 2.5, harvested by centrifugation, and lysed by incubation in 0.1 M NaOH for 5 min and boiling in SDS buffer (60 mM Tris–HCl, 2% SDS, 4% β-mercaptoethanol, 5% glycerol, 0.002% bromophenol blue) [1]. Samples were separated by 12% SDS-PAGE, blotted, and stained with 0.5% Ponceau S red (Sigma-Aldrich, Schnelldorf, Germany) in 1% acetic acid. After destaining, blots were incubated with anti-Myc-HRP (Abcam - ab62928, USA) in 1:5000 dilution for 1 h at room temperature. After washing in TBST buffer, chemiluminescence was detected using Super Signal™ West Dura Extended Duration Substrate (Thermo Fisher Scientific, Waltham, USA).

Protein modelling

Predictions of the 3D structures were created using AlphaFold3 [2]. The structures were visualized using the PyMOL software version 2.3.2 (Schrodinger Inc., USA).

1. Kushnirov, V.V. Rapid and reliable protein extraction from yeast. *Yeast* **2000**, *16*, 857-860, doi:10.1002/1097-0061(20000630)16:9<857::aid-yea561>3.0.co;2-b.
2. Abramson, J.; Adler, J.; Dunger, J.; Evans, R.; Green, T.; Pritzel, A.; Ronneberger, O.; Willmore, L.; Ballard, A.J.; Bambrick, J.; et al. Accurate structure prediction of biomolecular interactions with AlphaFold 3. *Nature* **2024**, *630*, 493-500, doi:10.1038/s41586-024-07487-w.
