# Supplementary material for: The Phenotype of Physcomitrium patens SMC6 Mutant with Interrupted Hinge Interactions
Source: Genes (Basel). 2025 Sep 16;16(9):1091. doi: 10.3390/genes16091091 (PMC12469541; doi:10.3390/genes16091091)
Supplement: Supplementary file 1 [file genes-16-01091-s001.zip › Supplement Table S1.pdf]

Table S1: Used oligonucleotides

Oligo		Purpose	
1459	GGTGGAGGACTTACAACCCATGATCCAATCGGTCCAAT GGGGTTTTAGAGCTATGCTGAA	PpSMC6-specific sgRNA spacer	F
1460	TTCAGCATAGCTCTAAAACCCCATTGGACCGATTGGATC ATGGGTTGTAAGTCCTCCACC	PpSMC6-specific sgRNA spacer	R
1461	TTCTCCATTCCGCCAATCGGACCGATTCGCTCCCATCTC ACCCTGGTTGATGCAACC	*G517R* mutation template	F
1462	GGTTGCATCAACCAGGGTGAGATGGGAGCGAATCGGTC CGATTGGCGGAATGGAGAA	*G517R* mutation template	R
1594	TTCTCCATTCCGCCAATCCGCCCGATTGGTTCCCATCTCA CCCTGGTTGATGCAACC	*G514R* mutation template	F
1595	GGTTGCATCAACCAGGGTGAGATGGGAACCAATCGGGC GGATTGGCGGAATGGAGAA	*G514R* mutation template	R
711	CCGTTAGCATCTCCACTAGG	genotyping	F
839	TGCACTCTTTCCACTGCCATTC	genotyping	R
1001	TACAATGCGGCTACCGAC	qPCR Cap-50	F
1002	AGGCCGGATGCAGTAAAC	qPCR Cap-50	R
Pp18S_A	CCTCTAAGAAGTTGGCCGCA	qPCR 18S rDNA	F
Pp18S_B	GGCCGTTCTTAGTTGGTGGA	qPCR 18S rDNA	R
Pp5S_F	TACCAAGGCTACTACACCAGATC	qPCR 5S rDNA	F
Pp5S_R	AGGTCACCCATCCCAGTACTA	qPCR 5S rDNA	R
ubqFw	ACTACCCTGAAGTTGTATAGTTCGG	qPCR ubiquitin	F
ubqRev	CAAGTCACATTACTTCGCTGTCTA	qPCR ubiquitin	R
